# Multimorbidity and Blood Pressure Control in 37 651 Hypertensive Patients From Danish General Practice

**DOI:** 10.1161/JAHA.112.004531

**Published:** 2013-02-22

**Authors:** Maja S. Paulsen, Morten Andersen, Janus L. Thomsen, Henrik Schroll, Pia V. Larsen, Jesper Lykkegaard, Ib A. Jacobsen, Mogens L. Larsen, Bo Christensen, Jens Sondergaard

**Affiliations:** 1 Research Unit of General Practice, Institute of Public Health, University of Southern Denmark, Odense, Denmark (M.S.P., M.A., J.L.T., P.V.L., J.L., J.S.); 2 Centre for Pharmacoepidemiology, Department of Medicine Solna, Karolinska Institutet, Stockholm, Sweden (M.A.); 3 Danish Quality Unit of General Practice, Odense, Denmark (J.L.T., H.S.); 4 The Clinic of Hypertension, Department of Endocrinology, Odense University Hospital, Odense, Denmark (I.A.J.); 5 Department of Cardiology, Odense University Hospital, Odense, Denmark (M.L.L.); 6 Department of General Practice, School of Public Health, University of Aarhus, Aarhus, Denmark (B.C.)

**Keywords:** cardiovascular diseases, comorbidities, hypertension, primary care

## Abstract

**Background:**

Patients with hypertension are primarily treated in general practice. However, major studies of patients with hypertension are rarely based on populations from primary care. Knowledge of blood pressure (BP) control rates in patients with diabetes and/or cardiovascular diseases (CVDs), who have additional comorbidities, is lacking. We aimed to investigate the association of comorbidities with BP control using a large cohort of hypertensive patients from primary care practices.

**Methods and Results:**

Using the Danish General Practice Database, we included 37 651 patients with hypertension from 231 general practices in Denmark. Recommended BP control was defined as BP <140/90 mm Hg in general and <130/80 mm Hg in patients with diabetes. The overall control rate was 33.2% (95% CI: 32.7 to 33.7). Only 16.5% (95% CI: 15.8 to 17.3) of patients with diabetes achieved BP control, whereas control rates ranged from 42.9% to 51.4% for patients with ischemic heart diseases or cerebrovascular or peripheral vascular diseases. A diagnosis of cardiac heart failure in addition to diabetes and/or CVD was associated with higher BP control rates, compared with men and women having only diabetes and/or CVD. A diagnosis of asthma in addition to diabetes and CVD was associated with higher BP control rates in men.

**Conclusion:**

In Danish general practice, only 1 of 3 patients diagnosed with hypertension had a BP below target. BP control rates differ substantially within comorbidities. Other serious comorbidities in addition to diabetes and/or CVD were not associated with lower BP control rates; on the contrary, in some cases the BP control rates were higher when the patient was diagnosed with other serious comorbidities in addition to diabetes and/or CVD.

## Introduction

Hypertension is a common disorder with an estimated prevalence of 22% to 27% worldwide, and it is an important risk factor for cardiovascular disease (CVD) such as stroke and myocardial infarction.^1–4^ Patients with hypertension are primarily treated in general practice, making it the most frequent reason for consultations with general practitioners (GPs).^5–6^ However, major studies of patients with hypertension are rarely based on populations from primary care. The importance of treating patients with hypertension to target blood pressure (BP) levels applies to patients with and without comorbidities. For example, patients with CVD have an increased risk of recurrent stroke or myocardial infarction if their BP is above recommended levels.^7–11^ Only a few studies exist on the prevalence of hypertension and rates of BP control among patients with CVD; these studies are mainly based on small selected populations^7,12–14^ or are not situated in primary care populations.^12–15^ In Europe, BP control rates in hypertensive patients range from 21% to 57%.^2,16–18^ The BP control rates are generally based on mixed populations of patients with and without comorbidities. Furthermore, patients with a chronic disease like hypertension often have 1 or more additional chronic comorbidity,^7^ and when patients have 2 or more comorbidities, the treatment of each of the comorbidities is generally poorer,^19–23^ which further affects a patient's chance of achieving BP control. To our knowledge, it has not been investigated how additional comorbidities are associated with BP control when patients already have diabetes and/or CVD. In this article, we aimed to analyze the association of comorbidities with BP control in a large cohort of hypertensive patients from primary care.

## Methods

### Study Design

From November 1, 2009, to January 31, 2011, we used the Danish General Practice Database (DAMD) to include 37 651 hypertensive patients from a sample of 231 general practices equally distributed across Denmark. From Statistics Denmark, we retrieved information about redeemed prescriptions, comorbidities, and number of contacts with the healthcare system.

### Danish Health Care

The healthcare system in Denmark is tax funded, providing universal access to general practice and hospital care for all inhabitants, regardless of age and geographic residence. A total of 98% of the 5.5 million Danish citizens are registered with a GP. There are ≈2100 general practices in Denmark representing ≈3780 GPs. Each GP has on average 1470 registered patients. The GPs are “gate‐keepers” for further contacts with the secondary healthcare system. Reimbursement for prescription medication increases with patient expenses.^24^

### Guidelines for Hypertension

GPs in Denmark have guidelines for BP measurement recommending that office BP should be measured at least twice with patients sitting down after 5 minutes of rest using a cuff properly adapted to the arm size. A maximum of a 5 mm Hg difference between the 2 last measurements is acceptable, and the mean of these 2 measurements is used for clinical decisions.^25–26^ The treatment goals are evidence based and recommended for patients up to the age of 80 years (BP below 140/90 mm Hg in general and <130/80 mm Hg in patients with diabetes).^25^ With home BP measurements, guidelines recommend BP <135/85 mm Hg in general and <130/80 mm Hg in patients with diabetes.

### Data Sources and Measurements

#### Danish General Practice Database

All inhabitants in Denmark can be identified by a unique civil registration number, allowing individual linkage across a vast number of registers. The DAMD contains information related to individual consultations with a GP.^27–28^ It uses a data capture module incorporated in the GP's information technology system. The data capture module automatically sends information on prescribed medication, diagnoses, disbursement codes, and laboratory data to DAMD for each contact with a patient. Diagnoses at contacts are coded according to the *International Classification of Primary Care* (ICPC) system (second edition).^29^ From each contact, we retrieved diagnoses, prescribed medication, and laboratory results including BP. By April 2012, a total of 52% of all practices in Denmark were registered in DAMD. A national agreement states that by April 2013, all 2100 practices in Denmark are obliged to use the data capture module and consequently contribute data to DAMD.

#### Statistics Denmark

The Danish Register of Medical Product Statistics contains all prescriptions since 1995 with patient identifier, date, and drug (anatomical therapeutic chemical [ATC] code). The Danish National Patient Register has information about admissions, outpatient services, and emergency department contacts with Danish hospitals since 1994 classified according to the *International Classification of Diseases, Tenth Revision*. The Danish Health Register has information on all contacts to the healthcare system since 1990.

#### Participants

**Figure 1.**
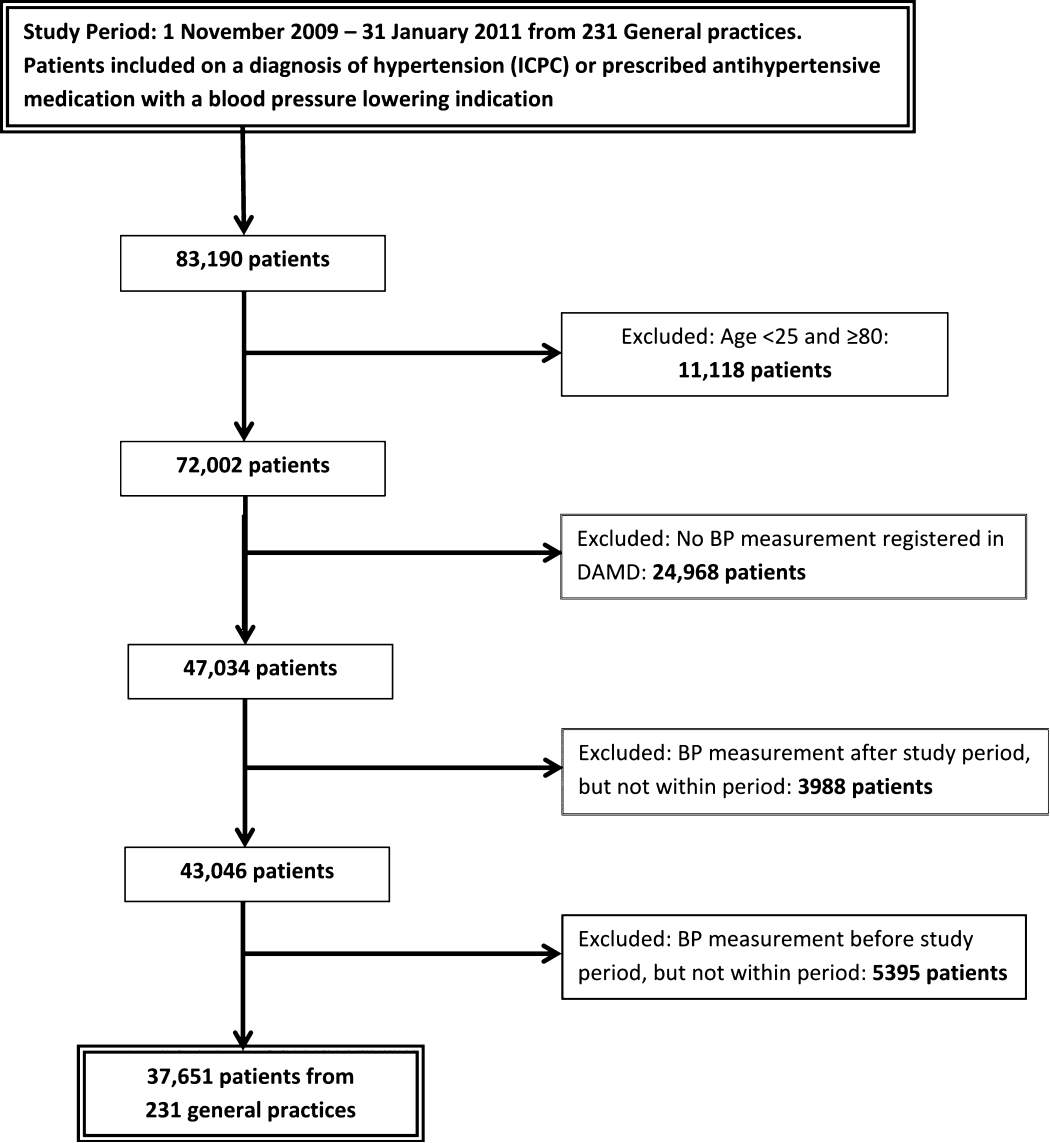
Flowchart. ICPC indicates International Classification of Primary Care; BP, blood pressure; DAMD, Danish General Practice Database.

Our study population was identified based on the following criteria: Patients were included if they had a consultation with their GP during the study period and if the reason for encounter was hypertension (ICPC diagnosis: K86; uncomplicated hypertension, K87; complicated hypertension) or if they were prescribed antihypertensive medication and the GP specifically wrote that the medication was BP‐lowering (ATC code C02‐C04; C06‐C09). These criteria indentified 83 190 patients with hypertension (Figure 1). In addition, patients had to be alive during the entire study period and registered with the same GP. Because treatment goals are recommended for patients up to the age of 80 years, patients <25 years and ≥80 years were excluded (11 118 patients). A total of 9383 patients had no registered BP measurement within the study period. An additional 24 968 patients had no BP measurement registered in DAMD. The final study population therefore consisted of 37 651 patients with hypertension from 231 practices (representing ≈415 GPs).

#### BP, index date, and time periods

The first BP measurement registered within the study period was used for analyses of BP control. The date of this measurement for each patient was defined as the index date. If 2 BP measurements were registered at the same index date, the mean of these was used for additional analyses. If there were >2 measurements, the mean of the 2 lowest measurements was used. Information from DAMD consisted of individual patient data 12 months before the index date. Information from the Danish National Patient Register and the Danish Register of Medical Product Statistics was available 15 years and 14 years before a patient's index date, respectively.

#### Antihypertensive drug treatment

Antihypertensive drug treatment included diuretics, angiotensin‐converting enzyme inhibitors (ACEIs), angiotensin II receptor blockers (ARBs), calcium channel blockers, β‐blockers, and other antihypertensive agents. To measure adherence, prescriptions redeemed before a patient's index date were used according to the following principle: 1 tablet covered a 1‐day supply (allowing for 20% nonadherence; eg, redemption of 100 tablets corresponded to 120 tablets). If a redeemed prescription provided a patient with enough tablets available to cover their index date, the patient was defined as being adherent to the redeemed drug. Combination drugs were split into each drug class. Patients were categorized as being adherent to treatment with 0, 1, 2, 3, or ≥4 drugs. The duration of antihypertensive treatment was categorized as follows: no prior prescription redeemed, 0 to 1 year, 1 to 2 years, 2 to 5 years, 5 to 10 years, or ≥10 years, based on the time interval between the index date and the first redemption of an antihypertensive drug prescription. We used DAMD to determine whether a GP had prescribed antihypertensive drugs to a patient.

#### Comorbidity

We defined patients as having diabetes based on a diagnosis of diabetes in DAMD or in the Danish National Patient Register or as having receiving prescribed or redeemed blood glucose–lowering drugs in the registers. From the Danish National Patient Register, we further extracted diagnoses for the following diseases: CVDs, ischemic heart diseases (IHDs), peripheral vascular diseases (PVDs), and other serious comorbidities (congestive heart failure [CHF], atrial fibrillation, chronic obstructive pulmony disease [COPD], osteoporosis, asthma, cancer, and psychiatric diseases).

### Statistical Analysis

The primary outcome measure is optimal BP control. Due to interaction between sex and age (*P*<0.001) and sex and CVD/diabetes comorbidities (*P*=0.0003) in relation to BP control, we stratified all tables and analyses according to sex. Patients were categorized as being diagnosed with either “no diabetes or CVD,” “CVD,” “diabetes,” or “diabetes plus CVD.” Means are presented with SDs in Table 1 and with 95% CIs around the means in Table 2. We used logistic regression to analyze the association between BP control and various covariates. All regression analyses were adjusted for cluster effect at practice level using robust cluster estimation. Odds ratios (ORs) are presented with 95% CIs. A value of *P<*.05 was considered statistically significant. We performed sensitivity analyses of BP control. In the first analysis, we used the lowest BP measurement instead of the mean of the 2 lowest measurements. In the second sensitivity analysis, we hypothesized that 57.4% of the 24 968 excluded patients (Figure 1) had achieved BP control. The 57.4% rate corresponds to control rates found in the most recent Danish study on hypertension.^2^ STATA release 12.0 (StataCorp, College Station, TX) was used for all statistical analyses.

**Table 1.** Characteristics of 37 651 Patients With Hypertension From Primary Care: BP, Treatment, and Comorbidities

	No. of Patients	Women*	Men*	All*
Participants	37 651	50.7 (n=19 107)	49.3 (n=18 544)	100 (n=37 651)
Age, mean y (SD)	37 651	63.6 (10.5)	62.9 (10.2)	63.3 (10.3)
BP, mm Hg				
Office systolic BP, mean (SD)	37 651	140.5 (17.4)	141.1 (17.3)	140.8 (17.4)
Office diastolic BP, mean (SD)	37 651	83.0 (10.7)	83.9 (11.1)	83.4 (10.9)
BP control	37 651	35.7	30.6	33.2
Duration of antihypertensive treatment				
No prescription redeemed	2304	5.1	7.2	6.1
0 to 1 year	2493	5.9	7.4	6.6
1 to 2 years	2140	5.2	6.2	5.7
2 to 5 years	6865	16.0	20.6	18.2
5 to 10 years	9405	24.2	25.8	25.0
≥10 years	14 444	43.7	32.8	38.4
Adherence to antihypertensive treatment*				
0 drugs	4923	12.5	13.7	13.1
1 drug	10 947	30.1	28.0	29.1
2 drugs	12 149	34.2	30.3	32.3
3 drugs	7351	18.3	20.8	18.5
≥4 drugs	2281	5.0	7.2	6.1
Comorbidity				
Diabetes*	9843	21.6	30.8	26.2
Diabetes*	7428	17.5	22.0	19.7
Diabetes plus CVD*	2415	4.1	8.8	6.4
CVD**	7170	14.0	24.3	19.0
Cerebrovascular disease*	3155	7.0	9.8	8.4
IHD*	3734	6.0	13.9	9.9
PVD*	1452	2.9	4.8	3.9
Other serious comorbidities*	9677	25.8	25.6	25.7
COPD	1610	4.5	4.1	4.3
Psychiatric diseases	2215	5.3	6.5	5.9
Asthma	1030	3.4	2.1	2.7
Osteoporosis	802	3.6	0.6	2.1
Cancer	3151	9.1	7.6	8.4
Congestive heart failure	1441	2.7	4.9	3.8
Atrial fibrillation	2251	4.2	7.8	5.6

BP indicates blood pressure; CVD, cardiovascular disease, IHD, ischemic heart disease; PVD, peripheral vascular disease; COPD, chronic obstructive pulmonary disease; GP, general practitioner; ACEI, angiotensin‐converting enzyme inhibitor; ARB, angiotensin receptor blocker.

* Percent, unless otherwise indicated as mean (SD) in first column.

* Number of antihypertensive drugs within the following: diuretics, ACEIs, ARBs, β‐blockers, or calcium channel blockers.

* Diagnosis of the disease, regardless of the presence of other diseases.

* Patients diagnosed with diabetes but without CVD. Patients could have a diagnosis of other serious comorbidities.

* Patients diagnosed with diabetes and CVD. Patients could have a diagnosis of other serious comorbidities.

* CVD includes cerebrovascular diseases, ISHs, and PVDs, where some patients have more than one of the diseases.

**Table 2.** BP Level and Control in Patients With and Without Comorbidities

	Women	Men	All
	BP control (%)*		
No comorbidity	39.5	32.4	37.0
Category for diabetes/CVD comorbidity*			
No CVD or diabetes*	40.0	33.9	37.4
CVD*	46.6	48.0	47.4
Diabetes	16.0	13.5	14.7
Diabetes plus CVD*	21.5	22.6	22.3
CVD without diabetes*			
Cerebrovascular disease	45.1	46.1	45.6
IHD	49.8	51.4	50.9
PVD	42.9	45.6	44.4
Other serious comorbidities*			
COPD	38.0	39.7	38.8
Psychiatric disease	38.1	35.3	35.0
Asthma	35.1	34.2	33.8
Osteoporosis	36.6	47.4	38.2
Cancer	34.8	35.8	35.2
Congestive heart failure	45.2	48.1	47.1
Atrial fibrillation	43.6	40.1	41.4
	BP levels, mean (95% CI)		
Category for diabetes/CVD comorbidity*			
No CVD or diabetes*	141.1 (141.1 to 141.7)	143.5 (143.1 to 143.8)	142.3 (142.1 to 142.5)
CVD*	139.2 (138.4 to 140.0)	138.0 (137.3 to 138.6)	138.5 (138.0 to 139.0)
Diabetes	138.3 (137.8 to 138.9)	139.6 (139.1 to 140.1)	139.0 (138.7 to 139.4)
Diabetes plus CVD*	137.0 (135.7 to 138.2)	135.9 (135.0 to 136.7)	136.2 (135.5 to 136.9)

BP indicates blood pressure; CVD, cardiovascular disease; IHD, ischemic heart disease; PVD, peripheral vascular disease; COPD, chronic obstructive pulmonary disease.

* BP control: BP <140/90 mm Hg in general and BP <130/80 mm Hg in patients diagnosed with diabetes.

* In the category for diabetes/CVD comorbidities, a patient was categorized as being diagnosed with either “no CVD or diabetes,” “CVD,” “diabetes,” or “diabetes plus CVD” regardless of the presence of other serious comorbidities.

* CVD includes cerebrovascular diseases, IHDs, and PVDs, where some patients have more than one of the diseases.

* Diagnosis of the disease, regardless of the presence of other diseases.

### Ethics

The study was approved by DAMD (10/09), the Danish Data Protection Agency (2009‐41‐4204), and the National Institute of Public Health (7‐604‐04‐2/139). The study did not need approval by the Regional Ethics Committee (http://www.dnvk.dk/CVK/Home/English.aspx).

## Results

Of the 37 651 patients included, 50.7% were women. Their mean±SD age was 63.3±10.3 years. A total of 22 595 patients (60.0%) were included due to a diagnosis of hypertension and the remaining 15 056 (40.0%) were included due to prescribed antihypertensive medication. Of those included due to a diagnosis of hypertension, 76.9% were prescribed antihypertensive drug treatment by their GP. Table 1 presents the baseline characteristics. More men than women were diagnosed with CVD (24.3% versus 14.0%) and with diabetes (30.8% versus 21.6%). The majority of patients (87.3%) had been treated for hypertension for >1 year and 63.4% had been treated for ≥5 years. A total of 6.1% had not redeemed a prescription for antihypertensive drugs during the past 14 years (Table 1).

### BP Control

**Figure 2.**
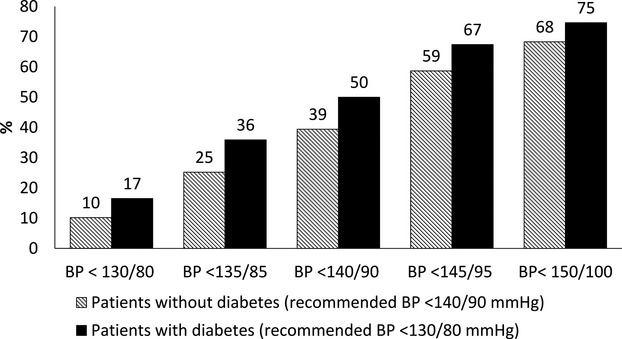
Proportion of patients with a blood pressure (BP) below the specified BP level.

The BP control rate for all 37 651 patients was 33.2% (95% CI: 32.7 to 33.7) (Table 1). Patients <40 years old had a control rate at 33.3%, whereas control rates in the other age groups were 40 to 49 years (29.9%), 50 to 59 years (32.3%), 60 to 69 years (33.5%), and 70 to 79 years (34.5%), respectively. For patients without diabetes 39% had a BP <140/90 mm Hg, whereas 59% had a BP <145/95 mm Hg (Figure 2).

### BP Control in Patients With Diabetes and CVD

In 9843 patients with diabetes an overall BP control rate of 16.5% (95% CI: 15.8 to 17.3) was achieved. A total of 50% of patients with diabetes had a BP <140/90 mm Hg, and 67% had BP <145/95 mm Hg (Figure 2). Table 2 presents BP control rates in patients with comorbidities. In patients with diabetes but without CVD, 14.7% (95% CI: 13.9 to 15.5) achieved BP control, whereas patients with diabetes and CVD had a higher control rate (22.3%; 95% CI: 20.6 to 23.9) (Table 2). The BP control rate for patients with CVD, but without diabetes, was 47.4% (95% CI: 46.0 to 48.9). Within cerebrovascular disease, myocardial infarction, and PVD, BP control rates ranged from 42.9% to 51.4% (Table 2).

### BP Control Within Other Serious Comorbidities

Patients diagnosed with congestive heart failure had the highest control rate at 47.1% (95% CI: 44.5 to 49.6), whereas patients having asthma or a psychiatric disease had the lowest at 33.8% (95% CI: 30.9 to 36.7) or 35.0% (95% CI: 33.0 to 37.0), respectively (Table 2). Generally, BP control rates did not differ much between sexes, except in patients diagnosed with osteoporosis (women: 36.6%, men: 47.4%) (Table 2).

### OR for BP Control in Patients With Diabetes and/or CVD

Women with CVD had higher odds of BP control (OR: 1.19 [95% CI: 1.09 to 1.31]) compared with women without diabetes or CVD (Table 3). However, women with diabetes (OR: 0.26 [95% CI: 0.23 to 0.30]) and women with diabetes plus CVD (OR: 0.35 [95% CI: 0.28 to 0.42]) had decreased odds of BP control (Table 3). The same statistically significant tendencies were seen for men, with OR for BP control at 1.39 (CVD), 0.26 (diabetes), and 0.40 (diabetes plus CVD), respectively, compared with men without diabetes or CVD (Table 3). CHF or atrial fibrillation was associated with higher odds of BP control for both men and women, whereas COPD only was associated with higher odds of BP control in men (OR: 1.25 [95% CI: 1.06 to 1.48]).

**Table 3.** OR for the Association Between BP Control and Comorbidities in 37 651 Patients

Specific Comorbidities	Adjusted for Age*	Adjusted for Age, Treatment, Duration, and Serious Comorbidities*	Adjusted for Age*	Adjusted for Age, Treatment, and Serious Comorbidities*
	Women		Men	
Category for diabetes/CVD comorbidity*				
No CVD or diabetes	1.00	1.00	1.00	1.00
CVD	1.35 (1.23 to 1.48)*	1.19 (1.09 to 1.31)*	1.65 (1.51 to 1.82)*	1.39 (1.26 to 1.53)*
Diabetes	0.29 (0.25 to 0.32)*	0.26 (0.23 to 0.30)*	0.30 (0.26 to 0.34)*	0.26 (0.23 to 0.30)*
Diabetes plus CVD	0.42 (0.35 to 0.51)*	0.35 (0.29 to 0.42)*	0.52 (0.44 to 0.61)*	0.40 (0.33 to 0.48)*
Serious comorbidities				
COPD	1.13 (0.96 to 1.34)	1.09 (0.92 to 1.31)	1.39 (1.20 to 1.61)*	1.25 (1.06 to 1.48)*
Psychiatric disease	1.11 (0.95 to 1.29)	1.14 (0.97 to 1.35)	1.13 (0.99 to 1.29)	1.06 (0.93 to 1.21)
Asthma	0.90 (0.77 to 1.05)	0.90 (0.77 to 1.06)	1.19 (0.96 to 1.47)	1.03 (0.81 to 1.31)
Osteoporosis	1.08 (0.92 to 1.27)	0.97 (0.81 to 1.14)	1.87 (1.33 to 2.62)*	1.39 (0.98 to 1.99)
Cancer	0.98 (0.87 to 1.10)	0.97 (0.85 to 1.11)	1.15 (1.04 to 1.28)*	1.07 (0.96 to 1.19)
Congestive heart failure	1.57 (1.30 to 1.90)*	1.54 (1.24 to 1.90)*	2.04 (1.80 to 2.32)*	1.90 (1.63 to 2.20)*
Atrial fibrillation	1.49 (1.28 to 1.72)*	1.34 (1.15 to 1.56)*	1.44 (1.30 to 1.60)*	1.19 (1.06 to 1.33)*

CVD indicates cardiovascular disease; COPD, chronic obstructive pulmonary disease; OR, odds ratio; BP, blood pressure.

* OR for the association between optimal BP control and each covariate, adjusted for age. ORs are presented with 95% CI.

* OR for the association between optimal BP control and CVD comorbidities adjusted for age, treatment (number of antihypertensive drugs), and each of the other serious comorbidities (asthma, COPD, cancer, osteoporosis, psychiatric diseases, atrial fibrillation, and congestive heart failure). ORs are presented with 95% CI.

* In the category for diabetes/CVD comorbidities, a patient was categorized as being diagnosed with either “no CVD or diabetes,” “CVD,” “diabetes,” or “diabetes plus CVD.”

* *P*<0.05.

* *P*<0.001.

* *P*<0.01.

### Patients Diagnosed With Serious Comorbidities in Addition to Diabetes and/or CVD

Table 4 present the proportions of patients with BP control for patients with various additional comorbidities, stratified on sex and diabetes and/or CVD comorbidities. If men with diabetes (BP control: 12.8%) had a further diagnosis of any of the other serious comorbidities, a higher proportion of patients achieved BP control. Examples were men with diabetes and COPD (BP control: 19.3%) and men with diabetes and atrial fibrillation (BP control: 17.4%). The same tendencies were seen for men having CVD and for men having diabetes and CVD (Table 4). A higher proportion of women achieved BP control if they had a diagnosis of COPD, CHF, and atrial fibrillation in addition to a diagnosis of diabetes and/or CVD (Table 4). However, in women diagnosed with CVD (BP control: 46.4%) a lower proportion achieved BP control, if they had one of the additional comorbidities psychiatric disease, asthma, osteoporosis, or cancer (Table 4). Table 5 shows the ORs of BP control for various additional comorbidities, stratified on sex and diabetes/CVD comorbidity. We found that a diagnosis of CHF in addition to diabetes and/or CVD was associated with higher odds of BP control for both men and women. A diagnosis of asthma in addition to diabetes and CVD was associated with higher odds of BP control in men. For both men and women, having a diagnosis of other serious comorbidities were never significantly associated with lower odds of BP control, if the patients also had diabetes and/or CVD (Table 5).

**Table 4.** Blood Pressure Control According to Comorbidities, Stratified on Sex, Diabetes, and/or CVD (Proportion of Patients With Blood Pressure Control—37 651 Patients)

Category for Diabetes/CVD Comorbidity*	No Diabetes or CVD (n=23 053)		Diabetes (n=7428)		CVD (n=4755)		Diabetes Plus CVD (n=2415)	
	Women	Men	Women	Men	Women	Men	Women	Men
No other serious comorbidities*	39.9	33.4	15.1	12.8	46.4	44.6	21.7	17.7
Other serious comorbidities								
COPD	42.0	42.0	20.1	19.3	49.7	55.5	23.2	36.1
Psychiatric disease	44.9	33.1	18.8	14.5	42.7	51.2	26.3	26.8
Asthma	38.3	35.6	14.8	15.2	44.7	46.4	25.0	43.6
Osteoporosis	38.7	43.4	13.4	33.3	43.7	59.5	29.0	45.5
Cancer	39.6	40.8	18.1	16.7	42.6	47.9	17.0	25.7
Congestive heart failure	52.8	53.2	35.4	23.6	56.0	67.3	26.3	35.4
Atrial fibrillation	53.2	46.3	23.9	17.4	47.5	55.7	24.2	28.3

CVD indicates cardiovascular disease; COPD, chronic obstructive pulmonary disease.

* In the category for CVD comorbidities, a patient was categorized as being diagnosed with either “no CVD or diabetes,” “CVD,” “diabetes,” or “diabetes plus CVD.”

* Other serious comorbidities include asthma, COPD, cancer, osteoporosis, psychiatric diseases, atrial fibrillation, and congestive heart failure.

**Table 5.** Odds Ratio for BP Control—Stratified on Sex, Diabetes, and/or CVD

Category for Diabetes/CVD Comorbidity*	No Diabetes or CVD* (n=23 053)	Diabetes* (n=7428)	CVD* (n=4755)	Diabetes Plus CVD* (n=2415)
Women	n=13 090	n=3345	n=1887	n=785
COPD	1.08 (0.88 to 1.31)	1.33 (0.90 to 1.98)	1.16 (0.84 to 1.61)	1.16 (0.84 to 1.62)
Psychiatric disease	1.23 (1.00 to 1.53)	1.27 (0.86 to 1.86)	0.86 (0.62 to 1.19)	1.29 (0.78 to 2.15)
Asthma	0.93 (0.76 to 1.13)	0.92 (0.54 to 1.55)	0.96 (0.62 to 1.49)	1.24 (0.56 to 2.75)
Osteoporosis	0.98 (0.81 to 1.18)	0.83 (0.40 to 1.68)	0.91 (0.63 to 1.31)	1.45 (0.73 to 2.86)
Cancer	0.99 (0.84 to 1.15)	1.19 (0.90 to 1.57)	0.86 (0.64 to 1.15)	0.74 (0.41 to 1.32)
Congestive heart failure	1.63 (1.18 to 2.25)*	2.87 (1.82 to 4.54)*	1.49 (1.05 to 2.11)*	1.31 (0.86 to 2.01)
Atrial fibrillation	1.63 (1.35 to 1.95)*	1.64 (1.09 to 2.47)*	1.05 (0.73 to 1.49)	1.22 (0.74 to 2.02)
Men	n=9963	n=4083	n=2868	n=1630
COPD	1.23 (0.97 to 1.55)	1.43 (0.96 to 2.15)	1.39 (1.04 to 1.85)*	1.84 (1.28 to 2.65)
Psychiatric disease	0.99 (0.83 to 1.18)	1.17 (0.82 to 1.66)	1.16 (0.91 to 1.49)	1.35 (0.92 to 1.99)
Asthma	1.06 (0.79 to 1.42)	1.14 (0.60 to 2.17)	0.95 (0.59 to 1.51)	2.72 (1.40 to 5.30)*
Osteoporosis	1.17 (0.72 to 1.89)	2.96 (1.06 to 8.33)	1.60 (0.82 to 3.11)	2.87 (0.88 to 9.38)
Cancer	1.15 (1.00 to 1.32)	1.15 (0.82 to 1.62)	0.94 (0.74 to 1.19)	1.13 (0.73 to 1.75)
Congestive heart failure	1.77 (1.32 to 2.37)*	1.91 (1.24 to 2.93)*	2.29 (1.74 to 3.01)*	2.01 (1.54 to 2.61)*
Atrial fibrillation	1.36 (1.15 to 1.60)*	1.27 (0.87 to 1.84)	1.38 (1.12 to 1.71)*	1.22 (0.88 to 1.70)

CVD indicates cardiovascular disease; COPD, chronic obstructive pulmonary disease; BP, blood pressure.

* In the category for CVD comorbidities, a patient was categorized as being diagnosed with either “no CVD or diabetes,” “CVD,” “diabetes,” or “diabetes plus CVD.”

* For each category of CVD comorbidities and for each sex, we used logistic regression to calculate the odds ratio for BP control adjusted for age, treatment (number of antihypertensive drugs), duration of antihypertensive drug treatment, and each of the other serious comorbidities (asthma, COPD, cancer, osteoporosis, psychiatric diseases, atrial fibrillation, and congestive heart failure).

* *P*<0.05.

* *P*<0.01.

* *P*<0.001.

### Sensitivity Analysis

In the first sensitivity analysis of BP control using the lowest BP measurement, overall BP control rates changed from 33.2% to 35.7%, and in patients with diabetes from 16.5% to 18.2%. In the second sensitivity analysis, where 57.4% of the 24 968 excluded patients were assumed to have achieved BP control, the estimated overall BP control rate would have increased to 42.8% if the 24 968 excluded patients had been included for analyses in the study population.

## Discussion

This study showed that only 33.2% of patients with hypertension treated in primary care in Denmark achieved BP control and that the BP control rate varied considerably between patients with comorbidities. Other serious comorbidities in addition to diabetes and/or CVD was not associated with lower odds of BP control, on the contrary, in some cases the odds of BP control were higher, when diagnosed with other serious comorbidities in addition to diabetes and/or CVD.

### BP Control

The low BP control rate found in our study is worrying, especially in patients with the highest need of BP control such as patients with diabetes or previous myocardial infarction or stroke. Although patients with CVD have a higher BP control rate than the overall study population, their control rates are still inadequate, because their risk of recurrent stroke or myocardial infarction increases with BP above recommended limits.^7,10,12,3^ However, raising the BP limit to 145/95 mm Hg increases the overall BP control rate from 39.1% to 58.7%, indicating that many patients are close to target BP levels (Figure 2, patients without diabetes). Some of this difference may be caused by “end‐digit preference,” which occurs when GPs round values up or down to multiples of 5 or 10.^30–32^ It could also be due to reluctance by the GP to intensify treatment when BP is close to target, for example, accepting a systolic value of 145 mm Hg, which is slightly above BP goal.^33–36^ A GP could have a resistance toward further medication due to lack of belief in additional BP‐lowering effect by adding a third or fourth antihypertensive drug. The patient might also resist further medication such as being treated with >5 different types of medications, which is not uncommon for patients with comorbidities.

Similar to other studies, we found that patients with diabetes have a much lower degree of BP control than the general population of patients with hypertension.^12,17–18,17–38^ The BP targets of BP <130/80 mm Hg in patients with diabetes may be more difficult to reach than BP limits <140/90 mm Hg, which is confirmed by the fact that 50% of all diabetics reached a BP <140/90 mm Hg (Figure 2). The BP control rate of 16.5% might even have been overestimated, because guidelines recommend that patients with diabetes and nephropathy should have a BP <120/80 mm Hg.^25^ Nephropathy is in general practice in Denmark measured through microalbuminuria, which we did not have information on. Furthermore, the ICPC system differentiates between diabetes type 1 and type 2 but not between diabetes with or without microalbuminuria. Hence, it was not possible to identify patients with diabetes and nephropathy and this could have overestimated their control rate. If patients with diabetes have BP limits below the general BP limits of 140/90 mm Hg, other priorities might influence the effort to lower BP down to the recommended 130/80 mm Hg. If a patient's BP was very high when treatment was started, it might be that a reduction of the BP to levels >130/80 mm Hg is considered acceptable.^33–34^ It is also possible that some GPs consider 140/90 mm Hg “close enough to target” or perhaps have a different perception of acceptable BP levels in patients with 2, 3, or more comorbidities. Because guidelines in Denmark and other countries are unambiguous, we need studies investigating barriers from GPs in their management of patients with hypertension.

### Comparison of BP Control Within Studies

We found that 33.2% of our study population achieved BP control. In a study by Gu et al,^15^ much higher control rates were found (women: 44.8%, men: 51.8%) in hypertensive patients from primary care. The proportion of patients with diabetes in their study was, however, much lower than in our study (men: 13.7%, women: 15.4%). In other studies of prevalence and control of hypertension, the proportion of patients with CVD and diabetes were even lower.^2,39^ Our study showed that comorbidity is an important aspect to consider because BP control rates range from 14.7% (diabetes without CVD) to 50.9% (IHD). Cautions are therefore in place regarding comparisons of BP control rates between studies with particular attention to comorbidity. For the organization of treatment and control of hypertension in primary care, the different BP control rates related to comorbidities are also important. Identifying patients whose BP is difficult to control may direct focus to the patients with the greatest need of attention. Our study population of hypertensive patients is included from general practices, which already have a data capture module installed in their medical information technology system. The data capture module provides the GPs with quality reports by which the GPs can identify their own hypertensive patients with and without comorbidities and with and without BP control. The extent to which GPs in these practices use the quality reports to improve treatment and control of hypertension is not yet investigated.

### Additional Comorbidities

Men diagnosed with asthma in addition to diabetes and CVD had higher odds of BP control compared with men having diabetes and CVD. This should be considered together with the fact that a patient with asthma needs treatment with β_2_‐agonist (bronchodilator) in periods with exacerbations. Asthma patients are therefore extra sensitive to treatment with β‐blocking agents for hypertension, because their asthma might become worse. Another side effect of β‐blocking agents is a rise in blood glucose levels in patients with diabetes, which may complicate the regulation of their diabetes. This illustrates that treating hypertensive patients with asthma, diabetes, and CVD is a major challenge and the fact that these patients had improved BP control is remarkable. Other serious comorbidities in addition to diabetes and/or CVD were not associated with statistical significantly lower odds of BP control. We believe that this could be due to the fact that patients with additional comorbidities have a higher frequency of contacts to their GPs than patients with no additional comorbidities. Although most comorbidity‐related consultations may not be planned for hypertension control with BP measurement, the frequent contacts to the GP may lead to a closer monitoring of BP, to better communication between GP and patient, to agreement on BP targets, and to an agreement on prevention of additional diseases.^40^ Furthermore, in Denmark, the GPs are responsible for treating almost all diseases and the GPs can therefore tailor a treatment strategy, taking all aspects of different diseases into consideration. Another reason for higher BP control rates in patients with additional comorbidities could be that patients with, for example, atrial fibrillation or CHF are treated with medications for their disease, which also lower their BP level. Patients with CHF in addition to diabetes and/or CVD were found to have a much higher degree of BP control in our study, which could be due to the tendency to falling BP in patients with CHF. In Denmark, there has been increased focus on the treatment of patients with psychiatric diseases and their comorbidities. It is generally considered that comorbidities in psychiatric patients are not treated adequately. Studies has furthermore reported that adherence to antihypertensive drug treatment in psychiatric patients is low, which influences a patient's ability to achieve BP control.^20,41^ However, hypertensive patients diagnosed with psychiatric diseases were not associated with lower BP control rate, if a patients had a psychiatric diseases in addition to diabetes and/or CVD. This may also be a consequence of psychiatric patients more frequent contact to their GPs. BP control rates in different comorbidities depends on many factors, hereby the medical treatments and the nature of each disease, which are complex interactions and will affect the patient's ability to achieve BP control.

### Strengths and Limitations

A main strength of this study is the inclusion of a large cohort of >37 000 patients with hypertension from primary care with no selection bias in relation to comorbidities and socioeconomic status. Furthermore, the inclusion did not depend on voluntary participation. A limitation is that we lack information on how the patients' BP was measured. However, the cross‐sectional design with inclusion of only one BP measurement was specifically chosen to resemble the “real world” of clinical practice, presenting a realistic picture of the hypertensive population as it is managed daily in primary care. The majority of patients (87.3%) had been treated with antihypertensive drugs for at least 1 year and their included BP measurement was presumably used to support the GPs in clinical decisions. The 6.1% of our study population who had not redeemed any prior prescriptions for antihypertensive drugs (Table 1) may be newly diagnosed with hypertension, be receiving nonpharmacological treatment, or be nonadherent for the past 15 years. Their BP levels have probably not reached target levels yet, because their regulation and intensifying of treatment are in an initial phase. The overall BP control rate only changed from 33.2% to 35.7% in the first sensitivity analysis using the lowest BP measurement instead of the mean of the 2 lowest measurements. Had we used a patient's last BP measurement registered in the study period, instead of the first BP measurement, it would have caused methodological problems: Patients included in the beginning of the study period would then have a longer observation time to improve their BP control compared with patients included at the end of the study period. By using the patient's first BP measurement, all patients have the same observations time to contribute a BP measurement. We excluded 24 968 patients from our study population because they had no BP measurement registered in DAMD (Figure 1, Table 6). The excluded 24 968 patients had the same distribution of age and sex as patients included in the study (Tables 1 and 6). However, fewer of the excluded patients had a diagnosis of diabetes. Depending on the procedures in each practice, some of the 24 968 patients could have had a BP measurement written in the medical records, which was not transferred to DAMD using the data capture module. Due to lower comorbidity rates, BP may not have been measured in the excluded patients, because they attended their GP for reasons other than hypertension. If 57.4% of all 24 968 patients achieved BP control, the sensitivity analysis showed that BP control rates increased from 33.2% to 42.8% if the excluded patients had been included in the study. Although this BP control rate is more acceptable, there is still room for improvement.

**Table 6.** Characteristics of the 37 651 Hypertensive Patients Included Compared With the 24 968 Excluded Patients Without a Blood Pressure Measurement Registered

Characteristics	Included Patients (%)*	Excluded Patients (%)*
N	37 651	24 968
Age, y*		
<40	931 (2.5)	758 (3.0)
40 to 49	3561 (9.5)	2825 (11.3)
50 to 59	8093 (21.5)	5899 (23.6)
60 to 69	14 083 (37.4)	9075 (36.4)
70 to 79	10 975 (29.2)	6411 (25.7)
Mean age, y (SD)	63.3 (10.3)	62.1 (10.4)
Sex		
Women	19 107 (50.8)	12 884 (51.6)
Men	18 544 (49.2)	12 084 (48.4)
Defined as hypertensive due to		
A diagnosis of hypertension	22 595 (60.0)	7069 (28.3)
Prescribed antihypertensive medication	15 056 (40.0)	17 899 (71.7)
Prescribed antihypertensive medication by their GP within the past year*	30 400 (80.7)	23 335 (93.5)
Contact with GP*		
No. of contacts with GP/y	9.63	7.69
Telephone contacts	2.73	2.67
Diagnosis of diabetes in DAMD*	9353 (24.8)	1870 (7.5)

BP indicates blood pressure; GP, general practitioner; DAMD, Danish General Practice Database.

* Number of contacts/year, age, prescribed antihypertensive medication by their GP, and a diagnosis of diabetes in DAMD were all measured according to a patient's index date. We had 1 year before a patient's index date available to investigate each covariate.

* For the included group of patients, their index date was the date of their first BP measurement present in DAMD. Because the 24 968 excluded patients had no BP measurement registered, we defined their index date to be the first date present of either a consultation with their GP or the date of prescribed antihypertensive medication, whichever were present first in our study period (November 1, 2009 to January 31, 2011).

### Generalizability

We included ≈84% of all patients with hypertension from our sample of 231 practices, assuming the true prevalence of hypertension is 25.7% and awareness of hypertension is 63.5% according to the most recent study of prevalence of hypertension in Denmark.^2^ One of our inclusion criteria was that patients had to attend their GP within a period of 15 months. Some patients attend their GP with longer time intervals, for example, every second or third year, probably because they have better BP control or no comorbidities. Had we extended our inclusion period and included patients attending their GP with wider time intervals, our BP control rate for patients without comorbidities might have been a little higher.

### Implications for Practice and Further Research

Further research should investigate differences in GPs' management of patients with and without comorbidities, and differences in patients' understanding and acceptance of treatment and control of hypertension with relation to comorbidities. Furthermore, it would be useful with more research targeting GPs' use of quality improvement reports in general and whether the use of these could lead to improvement in treatment and control of hypertensive patients.

## Conclusion

In Danish general practice only 1 of 3 patients diagnosed with hypertension had a BP below target. BP control rates differ substantially within comorbidities. BP control was poorer among patients with diabetes, whereas CVD in particular was associated with improved BP control. Other serious comorbidities in addition to diabetes and/or CVD was not associated with lower BP control rates; on the contrary, in some cases the BP control rates were higher, when diagnosed with other serious comorbidities in addition to diabetes and/or CVD.

## References

[1] Kearney PM Whelton M Reynolds K Whelton PK He J Worldwide prevalence of hypertension: a systematic review. J Hypertens. 2004; 22:11-19 15106785 10.1097/00004872-200401000-00003

[2] Kronborg CN Hallas J Jacobsen IA Prevalence, awareness, and control of arterial hypertension in Denmark. J Am Soc Hypertens. 2009; 3:19-24 20409941 10.1016/j.jash.2008.08.001

[3] Lewington S Clarke R Qizilbash N Peto R Collins R Age‐specific relevance of usual blood pressure to vascular mortality: a meta‐analysis of individual data for one million adults in 61 prospective studies. Lancet. 2002; 360:1903-1913 12493255 10.1016/s0140-6736(02)11911-8

[4] Turnbull F Effects of different blood‐pressure‐lowering regimens on major cardiovascular events: results of prospectively‐designed overviews of randomised trials. Lancet. 2003; 362:1527-1535 14615107 10.1016/s0140-6736(03)14739-3

[5] Moth G Vedsted P Olsen F Contact and Illness Pattern Study, KOS 2008, Aarhus. 2010 Aarhus, Denmark Research Unit for General Practice

[6] Munck A, Hansen DG. Audit on “Prevention in General Practice,” Answer Report. Total Results From 394 General Practitioners. Audit Projekt Odense (APO). Odense, Denmark: Research Unit for General Practice in Odense; 2005

[7] Kesarwani M Perez A Lopez VA Wong ND Franklin SS Cardiovascular comorbidities and blood pressure control in stroke survivors. J Hypertens. 2009; 27:1056-1063 19405168 10.1097/hjh.0b013e32832935ce

[8] Coca A Messerli FH Benetos A Zhou Q Champion A Cooper‐DeHoff RM Pepine CJ Predicting stroke risk in hypertensive patients with coronary artery disease: a report from the INVEST. Stroke. 2008; 39:343-348 18162623 10.1161/STROKEAHA.107.495465 PMC2805179

[9] Turnbull F Blood Pressure Lowering Treatment Trialists′ Collaboration Randomised trial of a perindopril‐based blood‐pressure‐lowering regimen among 6,105 individuals with previous stroke or transient ischaemic attack. Lancet. 2001; 358:1033-1041 11589932 10.1016/S0140-6736(01)06178-5

[10] Dorresteijn JA van der Graaf Y Spiering W Grobbee DE Bots ML Visseren FL Relation between blood pressure and vascular events and mortality in patients with manifest vascular disease: J‐curve revisited. Hypertension. 2012; 59:14-21 22068865 10.1161/HYPERTENSIONAHA.111.179143

[11] Turnbull F Neal B Algert C Chalmers J Chapman N Cutler J Woodward M MacMahon S Effects of different blood pressure‐lowering regimens on major cardiovascular events in individuals with and without diabetes mellitus: results of prospectively designed overviews of randomized trials. Arch Intern Med. 2005; 165:1410-1419 15983291 10.1001/archinte.165.12.1410

[12] Wong ND Lopez VA L'Italien G Chen R Kline SE Franklin SS Inadequate control of hypertension in US adults with cardiovascular disease comorbidities in 2003–2004. Arch Intern Med. 2007; 167:2431-2436 18071164 10.1001/archinte.167.22.2431

[13] Barrios V Escobar C Bertomeu V Murga N de Pablo C Calderon A Sex differences in the hypertensive population with chronic ischemic heart disease. J Clin Hypertens. 2008; 10:779-786 10.1111/j.1751-7176.2008.00020.x PMC8673085 19090879

[14] Barrios V Escobar C Calderon A Echarri R Gender differences in the management of diabetics patients with hypertension and chronic ischemic heart disease. Open Diabetes J. 2009; 2:1-4

[15] Gu Q Burt VL Paulose‐Ram R Dillon CF Gender differences in hypertension treatment, drug utilization patterns, and blood pressure control among US adults with hypertension: data from the National Health and Nutrition Examination Survey 1999–2004. Am J Hypertens. 2008; 21:789-798 18451806 10.1038/ajh.2008.185

[16] Paulsen MS Sondergaard J Reuther L Larsen PS Munck AP Larsen PV Damsgaard J Poulsen L Hansen DG Jacobsen IA Larsen ML Christensen HR Christensen B Andersen M Treatment of hypertension in general practice: a cross‐sectional study of 5413 hypertensive patients. Fam Pract. 2011; 28:599-607 21596691 10.1093/fampra/cmr027

[17] Barrios V Escobar C Calderon A Llisterri JL Echarri R Alegria E Muniz J Matali J Blood pressure and lipid goal attainment in the hypertensive population in the primary care setting in Spain. J Clin Hypertens. 2007; 9:324-329 10.1111/j.1524-6175.2007.06481.x PMC8109970 17485967

[18] Journath G Hellenius ML Petersson U Theobald H Nilsson PM Sex differences in risk factor control of treated hypertensives: a national primary healthcare‐based study in Sweden. Eur J Cardiovasc Prev Rehabil. 2008; 15:258-262 18525379 10.1097/HJR.0b013e3282f37a45

[19] Lund L Jacobsen J Norgaard M McLaughlin JK Blot WJ Borre M Sorensen HT The prognostic impact of comorbidities on renal cancer, 1995 to 2006: a Danish population based study. J Urol. 2009; 182:35-40 19450859 10.1016/j.juro.2009.02.136

[20] Bautista LE Vera‐Cala LM Colombo C Smith P Symptoms of depression and anxiety and adherence to antihypertensive medication. Am J Hypertens. 2012; 25:505-511 22258334 10.1038/ajh.2011.256 PMC3588114

[21] Soriano JB Visick GT Muellerova H Payvandi N Hansell AL Patterns of comorbidities in newly diagnosed COPD and asthma in primary care. Chest. 2005; 128:2099-2107 16236861 10.1378/chest.128.4.2099

[22] Piccirillo JF Vlahiotis A Comorbidity in patients with cancer of the head and neck: prevalence and impact on treatment and prognosis. Curr Oncol Rep. 2006; 8:123-129 16507222 10.1007/s11912-006-0047-z

[23] Corsonello A Antonelli Incalzi R Pistelli R Pedone C Bustacchini S Lattanzio F Comorbidities of chronic obstructive pulmonary disease. Curr Opin Pulm Med. 2011; 17suppl 1:S21-S28 22209926 10.1097/01.mcp.0000410744.75216.d0

[24] Moller PK Pricing and reimbursement of drugs in Denmark. Eur J Health Econ. 2003; 4:60-65 15609170 10.1007/s10198-003-0165-6

[25] The Danish Society of General Practitioners Christensen B Prevention of Ischemic Cardiovascular Disease in General Practice, 2007 Disease in General Practice. 2007 3rd edn Copenhagen, Denmark The Danish Society of General Practitioners

[26] Bang LE Diagnostic Blood Pressure Measurement—Diurnal, Home and Office Measurements. 2006 Copenhagen, Denmark The Danish Hypertension Society

[27] Schroll H Christensen B Andersen JS Sondergaard J Danish General Medicine Database—future tool! The Danish Society of General Medicine. Ugeskr Laeger. 2008; 170:1013 18397631

[28] Schroll H Christensen RD Thomsen JL Andersen M Friborg S Sondergaard J The Danish model for improvement of diabetes care in general practice: impact of automated collection and feedback of patient data. Int J Fam Med. 201210.1155/2012/208123 10.1155/2012/208123 PMC3409523 22888424

[29] Hofmans‐Okkes IM Lamberts H The International Classification of Primary Care (ICPC): new applications in research and computer‐based patient records in family practice. Fam Pract. 1996; 13:294-302 8671139 10.1093/fampra/13.3.294

[30] Bennett S Blood pressure measurement error: its effect on cross‐sectional and trend analyses. J Clin Epidemiol. 1994; 47:293-301 8138839 10.1016/0895-4356(94)90010-8

[31] Hense HW Kuulasmaa K Zaborskis A Kupsc W Tuomilehto J Quality assessment of blood pressure measurements in epidemiological surveys. The impact of last digit preference and the proportions of identical duplicate measurements. WHO Monica Project [corrected]. Rev Epidemiol Sante Publique. 1990; 38:463-468 2082452

[32] Nietert PJ Wessell AM Feifer C Ornstein SM Effect of terminal digit preference on blood pressure measurement and treatment in primary care. Am J Hypertens. 2006; 19:147-152 16448884 10.1016/j.amjhyper.2005.08.016

[33] Ferrari P Reasons for therapeutic inertia when managing hypertension in clinical practice in non‐Western countries. J Hum Hypertens. 2009; 23:151-159 18784735 10.1038/jhh.2008.117

[34] Ferrari P Hess L Pechere‐Bertschi A Muggli F Burnier M Reasons for not intensifying antihypertensive treatment (RIAT): a primary care antihypertensive intervention study. J Hypertens. 2004; 22:1221-1229 15167458 10.1097/00004872-200406000-00024

[35] Oliveria SA Lapuerta P McCarthy BD L'Italien GJ Berlowitz DR Asch SM Physician‐related barriers to the effective management of uncontrolled hypertension. Arch Intern Med. 2002; 162:413-420 11863473 10.1001/archinte.162.4.413

[36] Bramlage P Thoenes M Kirch W Lenfant C Clinical practice and recent recommendations in hypertension management–reporting a gap in a global survey of 1259 primary care physicians in 17 countries. Curr Med Res Opin. 2007; 23:783-791 17407635 10.1185/030079907x182077

[37] Qvarnstrom M Wettermark B Ljungman C Zarrinkoub R Hasselstrom J Manhem K Sundström A Kahan T Antihypertensive treatment and control in a large primary care population of 21 167 patients. J Hum Hypertens. 2011; 25:484-491 20720572 10.1038/jhh.2010.86

[38] Tabenkin H Eaton CB Roberts MB Parker DR McMurray JH Borkan J Differences in cardiovascular disease risk factor management in primary care by sex of physician and patient. Ann Fam Med. 2010; 8:25-32 20065275 10.1370/afm.1071 PMC2807384

[39] Ong KL Cheung BM Man YB Lau CP Lam KS Prevalence, awareness, treatment, and control of hypertension among United States adults 1999–2004. Hypertension. 2007; 49:69-75 17159087 10.1161/01.HYP.0000252676.46043.18

[40] Naik AD Kallen MA Walder A Street RL Jr Improving hypertension control in diabetes mellitus: the effects of collaborative and proactive health communication. Circulation. 2008; 117:1361-1368 18316489 10.1161/CIRCULATIONAHA.107.724005 PMC3717409

[41] Krousel‐Wood MA Frohlich ED Hypertension and depression: coexisting barriers to medication adherence. J Clin Hypertens. 2010; 12:481-486 10.1111/j.1751-7176.2010.00302.x PMC3050602 20629809

